# Supplementary material for: The effectiveness of organisational-level workplace mental health interventions on mental health and wellbeing in construction workers: A systematic review and recommended research agenda
Source: PLoS One. 2022 Nov 16;17(11):e0277114. doi: 10.1371/journal.pone.0277114 (PMC9668198; doi:10.1371/journal.pone.0277114)
Supplement: S1 File — (PDF) [file pone.0277114.s002.pdf]

MY EBSCOhost

Thursday, March 18, 2021 5:54:38 AM

#	Query	Limiters/Expanders	Last Run Via	Results
S29	S6 AND S16 AND S26	Limiters - Published Date: 20100101-20211231<br>Expanders - Apply equivalent subjects<br>Narrow by Language: - english<br>Search modes - Boolean/Phrase	Interface - EBSCOhost Research Databases<br>Search Screen - Advanced Search<br>Database - Academic Search Complete	247
S28	S6 AND S16 AND S26	Limiters - Published Date: 20100101-20211231<br>Expanders - Apply equivalent subjects<br>Search modes - Boolean/Phrase	Interface - EBSCOhost Research Databases<br>Search Screen - Advanced Search<br>Database - Academic Search Complete	269
S27	S6 AND S16 AND S26	Expanders - Apply equivalent subjects<br>Search modes - Boolean/Phrase	Interface - EBSCOhost Research Databases<br>Search Screen - Advanced Search<br>Database - Academic Search Complete	321
S26	S17 OR S18 OR S19 OR S20 OR S21 OR S22 OR S23 OR S24 OR S25	Expanders - Apply equivalent subjects<br>Search modes - Boolean/Phrase	Interface - EBSCOhost Research Databases<br>Search Screen - Advanced Search<br>Database - Academic Search Complete	476,173
S25	TI (depressive symptoms or anxiety symptoms or stress symptoms or burnout) OR AB (depressive symptoms or anxiety symptoms or stress symptoms or burnout)	Expanders - Apply equivalent subjects<br>Search modes - Boolean/Phrase	Interface - EBSCOhost Research Databases<br>Search Screen - Advanced Search<br>Database - Academic Search Complete	84,254
S24	TI (psychological health or mental health) OR AB (psychological health or mental health)	Expanders - Apply equivalent subjects<br>Search modes - Boolean/Phrase	Interface - EBSCOhost Research Databases<br>Search Screen - Advanced Search	177,229

			Database - Academic Search Complete	
S23	TI (mental well-being or mental wellness or psychological well-being) OR AB (mental well-being or mental wellness or psychological well-being)	Expanders - Apply equivalent subjects<br>Search modes - Boolean/Phrase	Interface - EBSCOhost Research Databases<br>Search Screen - Advanced Search<br>Database - Academic Search Complete	16,396
S22	TI (wellbeing or well-being or well being) OR AB (wellbeing or well-being or well being)	Expanders - Apply equivalent subjects<br>Search modes - Boolean/Phrase	Interface - EBSCOhost Research Databases<br>Search Screen - Advanced Search<br>Database - Academic Search Complete	119,679
S21	SU psychological well-being	Expanders - Apply equivalent subjects<br>Search modes - Boolean/Phrase	Interface - EBSCOhost Research Databases<br>Search Screen - Advanced Search<br>Database - Academic Search Complete	3,683
S20	SU burnout	Expanders - Apply equivalent subjects<br>Search modes - Boolean/Phrase	Interface - EBSCOhost Research Databases<br>Search Screen - Advanced Search<br>Database - Academic Search Complete	8,126
S19	SU anxiety	Expanders - Apply equivalent subjects<br>Search modes - Boolean/Phrase	Interface - EBSCOhost Research Databases<br>Search Screen - Advanced Search<br>Database - Academic Search Complete	75,589
S18	SU mental depression	Expanders - Apply equivalent subjects<br>Search modes - Boolean/Phrase	Interface - EBSCOhost Research Databases<br>Search Screen - Advanced Search<br>Database - Academic Search Complete	112,091
S17	SU job stress	Expanders - Apply equivalent subjects<br>Search modes - Boolean/Phrase	Interface - EBSCOhost Research Databases<br>Search Screen - Advanced Search<br>Database - Academic Search Complete	12,957

S16	S10 AND S15	Expanders - Apply equivalent subjects<br>Search modes - Boolean/Phrase	Interface - EBSCOhost Research Databases<br>Search Screen - Advanced Search<br>Database - Academic Search Complete	495,635
S15	S11 OR S12 OR S13 OR S14	Expanders - Apply equivalent subjects<br>Search modes - Boolean/Phrase	Interface - EBSCOhost Research Databases<br>Search Screen - Advanced Search<br>Database - Academic Search Complete	2,926,643
S14	TI (worksite or work site or job site or office) OR AB (worksite or work site or job site or office)	Expanders - Apply equivalent subjects<br>Search modes - Boolean/Phrase	Interface - EBSCOhost Research Databases<br>Search Screen - Advanced Search<br>Database - Academic Search Complete	233,880
S13	TI (organization* or organisation*) OR AB (organization* or organisation*)	Expanders - Apply equivalent subjects<br>Search modes - Boolean/Phrase	Interface - EBSCOhost Research Databases<br>Search Screen - Advanced Search<br>Database - Academic Search Complete	683,156
S12	TI (work or workplace or work place) OR AB (work or workplace or work place)	Expanders - Apply equivalent subjects<br>Search modes - Boolean/Phrase	Interface - EBSCOhost Research Databases<br>Search Screen - Advanced Search<br>Database - Academic Search Complete	2,125,356
S11	SU work environment	Expanders - Apply equivalent subjects<br>Search modes - Boolean/Phrase	Interface - EBSCOhost Research Databases<br>Search Screen - Advanced Search<br>Database - Academic Search Complete	37,268
S10	S7 OR S8 OR S9	Expanders - Apply equivalent subjects<br>Search modes - Boolean/Phrase	Interface - EBSCOhost Research Databases<br>Search Screen - Advanced Search<br>Database - Academic Search Complete	3,566,585
S9	TI (intervention* or program* or prevention or training or initiative* or	Expanders - Apply equivalent subjects	Interface - EBSCOhost Research Databases<br>Search Screen - Advanced	3,566,179

	policy or promotion or campaign*) OR AB (intervention* or program* or prevention or training or initiative* or policy or promotion or campaign*)	Search modes - Boolean/Phrase	Search<br>Database - Academic Search Complete	
S8	SU mental health promotion	Expanders - Apply equivalent subjects<br>Search modes - Boolean/Phrase	Interface - EBSCOhost Research Databases<br>Search Screen - Advanced Search<br>Database - Academic Search Complete	700
S7	SU program implementation	Expanders - Apply equivalent subjects<br>Search modes - Boolean/Phrase	Interface - EBSCOhost Research Databases<br>Search Screen - Advanced Search<br>Database - Academic Search Complete	689
S6	S1 OR S2 OR S3 OR S4 OR S5	Expanders - Apply equivalent subjects<br>Search modes - Boolean/Phrase	Interface - EBSCOhost Research Databases<br>Search Screen - Advanced Search<br>Database - Academic Search Complete	254,733
S5	TI ("construction worker*" or "civil engineer*" or laborer* or labourer* or carpenter* or electrician* or builder* or painter* or plumber* or "heavy equipment operator*" or plasterer* or mason* or pipefitter* or "iron worker*") OR AB ("construction worker*" or "civil engineer*" or laborer* or labourer* or carpenter* or electrician* or builder* or painter* or plumber* or "heavy equipment operator*" or plasterer* or mason* or pipefitter* or "iron worker*")	Expanders - Apply equivalent subjects<br>Search modes - Boolean/Phrase	Interface - EBSCOhost Research Databases<br>Search Screen - Advanced Search<br>Database - Academic Search Complete	87,319
S4	SU construction industry	Expanders - Apply equivalent subjects	Interface - EBSCOhost Research Databases	25,392

		Search modes - Boolean/Phrase	Search Screen - Advanced Search<br>Database - Academic Search Complete	
S3	AB Building AND AB (industr* or sector* or trade or business* or compan*)	Expanders - Apply equivalent subjects<br>Search modes - Boolean/Phrase	Interface - EBSCOhost Research Databases<br>Search Screen - Advanced Search<br>Database - Academic Search Complete	54,193
S2	AB Construction AND AB (industr* or sector* or trade or business* or compan*)	Expanders - Apply equivalent subjects<br>Search modes - Boolean/Phrase	Interface - EBSCOhost Research Databases<br>Search Screen - Advanced Search<br>Database - Academic Search Complete	60,606
S1	TI Construction	Expanders - Apply equivalent subjects<br>Search modes - Boolean/Phrase	Interface - EBSCOhost Research Databases<br>Search Screen - Advanced Search<br>Database - Academic Search Complete	69,784

PubMed Search Results

Search number	Query	Filters	Results
32	#30 and #6 and #16	Full text, Humans, English, from 2010 - 2020	55
31	#30 and #6 and #16	Full text, Humans, English	74
30	#17 or #18 or #19 or #20 or #21 or #22 or #23 or #24 or #25 or #26 or #27 or #28 or #29	Full text, Humans, English	270,467
29	burnout[Title/Abstract] OR burn out[Title/Abstract]	Full text, Humans, English	7,001
28	depressive symptoms[Title/Abstract] OR anxiety symptoms[Title/Abstract] OR stress symptoms[Title/Abstract]	Full text, Humans, English	43,646
27	psychological wellbeing[Title/Abstract] or psychological well-being[Title/Abstract] or psychological well being[Title/Abstract]	Full text, Humans, English	7,126
26	psychological health[Title/Abstract]	Full text, Humans, English	3,497
25	mental wellness[Title/Abstract]	Full text, Humans, English	67
24	mental well being[Title/Abstract] OR mental well-being[Title/Abstract] OR mental wellbeing[Title/Abstract]	Full text, Humans, English	1,952
23	well being[Title/Abstract] OR well-being[Title/Abstract] OR wellbeing[Title/Abstract]	Full text, Humans, English	58,804
22	occupational stress[MeSH Terms]	Full text, Humans, English	9,169
21	burnout, psychological[MeSH Terms]	Full text, Humans, English	8,091
20	burnout, professional[MeSH Terms]	Full text, Humans, English	7,926
19	anxiety[MeSH Terms]	Full text, Humans, English	57,443
18	depression[MeSH Terms]	Full text, Humans, English	163,888
17	depressive disorder[MeSH Terms]	Full text, Humans, English	84,193
16	#10 and #15	Full text, Humans, English	136,190
15	#11 or #12 or #13 or #14	Full text, Humans, English	426,433
14	organization*[Title/Abstract] OR organisation*[Title/Abstract]	Full text, Humans, English	193,161

13	worksite*[Title/Abstract] OR work site*[Title/Abstract] OR job site*[Title/Abstract] OR office*[Title/Abstract]	Full text, Humans, English	49,734
12	organizations[MeSH Terms]	Full text, Humans, English	199,036
11	workplace[MeSH Terms]	Full text, Humans, English	17,135
10	#7 or #8 or #9	Full text, Humans, English	1,554,562
9	secondary prevention[MeSH Terms]	Full text, Humans, English	15,814
8	primary prevention[MeSH Terms]	Full text, Humans, English	66,300
7	intervention*[Title/Abstract] OR program*[Title/Abstract] OR prevention[Title/Abstract] OR training[Title/Abstract] OR initiative*[Title/Abstract] OR policy[Title/Abstract] OR promotion[Title/Abstract] OR campaign*[Title/Abstract]	Full text, Humans, English	1,504,698
6	#1 or #2 or #3 or #4 or #5	Full text, Humans, English	16,806
5	"construction worker*"[Title/Abstract] OR "civil engineer*"[Title/Abstract] OR laborer*[Title/Abstract] OR labourer*[Title/Abstract] OR carpenter*[Title/Abstract] OR electrician*[Title/Abstract] OR builder*[Title/Abstract] OR painter*[Title/Abstract] OR plumber*[Title/Abstract] OR "heavy equipment operator*"[Title/Abstract] OR plasterer*[Title/Abstract] OR mason*[Title/Abstract] OR pipefitter*[Title/Abstract] OR "iron worker*"[Title/Abstract]	Full text, Humans, English	5,885
4	Construction[Title]	Full text, Humans, English	7,215
3	Building[Title/Abstract] AND (industr*[Title/Abstract] OR sector*[Title/Abstract] OR compan*[Title/Abstract] OR business*[Title/Abstract] OR trade*)[Title/Abstract]	Full text, Humans, English	2,935

2	Construction[Title/Abstract] AND (industr*[Title/Abstract] OR sector*[Title/Abstract] OR compan*[Title/Abstract] OR business*[Title/Abstract] OR trade*)[Title/Abstract]	Full text, Humans, English	2,468
1	construction industry[MeSH Terms]	Full text, Humans, English	994

MY EBSCOhost

Thursday, March 18, 2021 6:02:33 AM

#	Query	Limiters/Expanders	Last Run Via	Results
S40	S18 AND S30 AND S37	Limiters - Publication Year: 2010-2021<br>Expanders - Apply equivalent subjects<br>Narrow by Language: - english<br>Search modes - Boolean/Phrase	Interface - EBSCOhost Research Databases<br>Search Screen - Advanced Search<br>Database - APA PsycInfo	99
S39	S18 AND S30 AND S37	Limiters - Publication Year: 2010-2021<br>Expanders - Apply equivalent subjects<br>Search modes - Boolean/Phrase	Interface - EBSCOhost Research Databases<br>Search Screen - Advanced Search<br>Database - APA PsycInfo	170
S38	S18 AND S30 AND S37	Expanders - Apply equivalent subjects<br>Search modes - Boolean/Phrase	Interface - EBSCOhost Research Databases<br>Search Screen - Advanced Search<br>Database - APA PsycInfo	233
S37	S1 OR S2 OR S3 OR S36	Expanders - Apply equivalent subjects<br>Search modes - Boolean/Phrase	Interface - EBSCOhost Research Databases<br>Search Screen - Advanced Search<br>Database - APA PsycInfo	26,563
S36	S34 OR S35	Expanders - Apply equivalent subjects<br>Search modes - Boolean/Phrase	Interface - EBSCOhost Research Databases<br>Search Screen - Advanced Search<br>Database - APA PsycInfo	4,562
S35	AB ("construction worker*" or "civil engineer*" or carpenter* or electrician* or builder* or painter* or plumber* or "heavy equipment operator*" or plasterer* or mason* or pipefitter* or "iron worker*")	Expanders - Apply equivalent subjects<br>Search modes - Boolean/Phrase	Interface - EBSCOhost Research Databases<br>Search Screen - Advanced Search<br>Database - APA PsycInfo	4,345
S34	TI ("construction	Expanders - Apply	Interface - EBSCOhost	644

	worker*" or "civil engineer*" or carpenter* or electrician* or builder* or painter* or plumber* or "heavy equipment operator*" or plasterer* or mason* or pipefitter* or "iron worker*")	equivalent subjects<br>Search modes - Boolean/Phrase	Research Databases<br>Search Screen - Advanced Search<br>Database - APA PsycInfo	
S33	S4 AND S18 AND S30	Limiters - Publication Year: 2010-2020<br>Expanders - Apply equivalent subjects<br>Search modes - Boolean/Phrase	Interface - EBSCOhost Research Databases<br>Search Screen - Advanced Search<br>Database - APA PsycInfo	154
S32	S4 AND S18 AND S30	Limiters - Publication Year: 2010-2020<br>Expanders - Apply equivalent subjects<br>Search modes - Boolean/Phrase	Interface - EBSCOhost Research Databases<br>Search Screen - Advanced Search<br>Database - APA PsycInfo	154
S31	S4 AND S18 AND S30	Expanders - Apply equivalent subjects<br>Search modes - Boolean/Phrase	Interface - EBSCOhost Research Databases<br>Search Screen - Advanced Search<br>Database - APA PsycInfo	210
S30	S19 OR S20 OR S21 OR S22 OR S23 OR S24 OR S25 OR S26 OR S27 OR S28 OR S29	Expanders - Apply equivalent subjects<br>Search modes - Boolean/Phrase	Interface - EBSCOhost Research Databases<br>Search Screen - Advanced Search<br>Database - APA PsycInfo	487,779
S29	TI (burnout or burn-out or burn out) OR AB (burnout or burn-out or burn out)	Expanders - Apply equivalent subjects<br>Search modes - Boolean/Phrase	Interface - EBSCOhost Research Databases<br>Search Screen - Advanced Search<br>Database - APA PsycInfo	13,212
S28	TI (depressive symptoms or anxiety symptoms or stress symptoms) OR AB (depressive symptoms or anxiety symptoms or stress symptoms)	Expanders - Apply equivalent subjects<br>Search modes - Boolean/Phrase	Interface - EBSCOhost Research Databases<br>Search Screen - Advanced Search<br>Database - APA PsycInfo	85,732
S27	TI (psychological health or mental health) OR AB	Expanders - Apply equivalent subjects	Interface - EBSCOhost Research Databases<br>Search Screen - Advanced	205,961

	(psychological health or mental health)	Search modes - Boolean/Phrase	Search<br>Database - APA PsycInfo	
S26	TI (mental well-being or mental wellness or psychological well-being) OR AB (mental well-being or mental wellness or psychological well-being)	Expanders - Apply equivalent subjects<br>Search modes - Boolean/Phrase	Interface - EBSCOhost Research Databases<br>Search Screen - Advanced Search<br>Database - APA PsycInfo	20,663
S25	DE well being	Expanders - Apply equivalent subjects<br>Search modes - Boolean/Phrase	Interface - EBSCOhost Research Databases<br>Search Screen - Advanced Search<br>Database - APA PsycInfo	46,881
S24	DE mental health	Expanders - Apply equivalent subjects<br>Search modes - Boolean/Phrase	Interface - EBSCOhost Research Databases<br>Search Screen - Advanced Search<br>Database - APA PsycInfo	77,043
S23	DE positive psychology	Expanders - Apply equivalent subjects<br>Search modes - Boolean/Phrase	Interface - EBSCOhost Research Databases<br>Search Screen - Advanced Search<br>Database - APA PsycInfo	4,902
S22	DE Occupational stress	Expanders - Apply equivalent subjects<br>Search modes - Boolean/Phrase	Interface - EBSCOhost Research Databases<br>Search Screen - Advanced Search<br>Database - APA PsycInfo	22,313
S21	DE Stress	Expanders - Apply equivalent subjects<br>Search modes - Boolean/Phrase	Interface - EBSCOhost Research Databases<br>Search Screen - Advanced Search<br>Database - APA PsycInfo	64,643
S20	DE Anxiety	Expanders - Apply equivalent subjects<br>Search modes - Boolean/Phrase	Interface - EBSCOhost Research Databases<br>Search Screen - Advanced Search<br>Database - APA PsycInfo	83,887
S19	DE Depression	Expanders - Apply equivalent subjects<br>Search modes - Boolean/Phrase	Interface - EBSCOhost Research Databases<br>Search Screen - Advanced Search<br>Database - APA PsycInfo	47,811

S18	S13 AND S17	Expanders - Apply equivalent subjects<br>Search modes - Boolean/Phrase	Interface - EBSCOhost Research Databases<br>Search Screen - Advanced Search<br>Database - APA PsycInfo	227,583
S17	S14 OR S15 OR S16	Expanders - Apply equivalent subjects<br>Search modes - Boolean/Phrase	Interface - EBSCOhost Research Databases<br>Search Screen - Advanced Search<br>Database - APA PsycInfo	753,561
S16	TI (worksite or work site or job site or office) OR AB (worksite or work site or job site or office)	Expanders - Apply equivalent subjects<br>Search modes - Boolean/Phrase	Interface - EBSCOhost Research Databases<br>Search Screen - Advanced Search<br>Database - APA PsycInfo	25,432
S15	TI (organization or organisation or organizational or organisational) OR AB (organization or organisation or organizational or organisational)	Expanders - Apply equivalent subjects<br>Search modes - Boolean/Phrase	Interface - EBSCOhost Research Databases<br>Search Screen - Advanced Search<br>Database - APA PsycInfo	270,330
S14	TI (work or workplace or work place) OR AB (work or workplace or work place)	Expanders - Apply equivalent subjects<br>Search modes - Boolean/Phrase	Interface - EBSCOhost Research Databases<br>Search Screen - Advanced Search<br>Database - APA PsycInfo	539,732
S13	S5 OR S6 OR S7 OR S8 OR S9 OR S10 OR S11 OR S12	Expanders - Apply equivalent subjects<br>Search modes - Boolean/Phrase	Interface - EBSCOhost Research Databases<br>Search Screen - Advanced Search<br>Database - APA PsycInfo	1,099,446
S12	TI (intervention* or program* or prevention or training or initiative* or policy or promotion or campaign*) OR AB (intervention* or program* or prevention or training or initiative* or policy or promotion or campaign*)	Expanders - Apply equivalent subjects<br>Search modes - Boolean/Phrase	Interface - EBSCOhost Research Databases<br>Search Screen - Advanced Search<br>Database - APA PsycInfo	1,090,185
S11	DE primary mental health prevention	Expanders - Apply equivalent subjects	Interface - EBSCOhost Research Databases<br>Search Screen - Advanced	0

		Search modes - Boolean/Phrase	Search<br>Database - APA PsycInfo	
S10	DE occupational health psychology	Expanders - Apply equivalent subjects<br>Search modes - Boolean/Phrase	Interface - EBSCOhost Research Databases<br>Search Screen - Advanced Search<br>Database - APA PsycInfo	163
S9	DE prevention	Expanders - Apply equivalent subjects<br>Search modes - Boolean/Phrase	Interface - EBSCOhost Research Databases<br>Search Screen - Advanced Search<br>Database - APA PsycInfo	31,813
S8	DE mental health program evaluation	Expanders - Apply equivalent subjects<br>Search modes - Boolean/Phrase	Interface - EBSCOhost Research Databases<br>Search Screen - Advanced Search<br>Database - APA PsycInfo	2,174
S7	DE program evaluation	Expanders - Apply equivalent subjects<br>Search modes - Boolean/Phrase	Interface - EBSCOhost Research Databases<br>Search Screen - Advanced Search<br>Database - APA PsycInfo	24,278
S6	DE workplace intervention	Expanders - Apply equivalent subjects<br>Search modes - Boolean/Phrase	Interface - EBSCOhost Research Databases<br>Search Screen - Advanced Search<br>Database - APA PsycInfo	977
S5	DE intervention	Expanders - Apply equivalent subjects<br>Search modes - Boolean/Phrase	Interface - EBSCOhost Research Databases<br>Search Screen - Advanced Search<br>Database - APA PsycInfo	69,009
S4	S1 OR S2 OR S3	Expanders - Apply equivalent subjects<br>Search modes - Boolean/Phrase	Interface - EBSCOhost Research Databases<br>Search Screen - Advanced Search<br>Database - APA PsycInfo	22,387
S3	AB Building AND AB (industr* or sector* or compan* or business* or trade*)	Expanders - Apply equivalent subjects<br>Search modes - Boolean/Phrase	Interface - EBSCOhost Research Databases<br>Search Screen - Advanced Search<br>Database - APA PsycInfo	6,404
S2	AB Construction AND AB (industr* or sector* or	Expanders - Apply equivalent subjects	Interface - EBSCOhost Research Databases	3,719

	compan* or business* or trade*)	Search modes - Boolean/Phrase	Search Screen - Advanced Search<br>Database - APA PsycInfo	
S1	TI Construction	Expanders - Apply equivalent subjects<br>Search modes - Boolean/Phrase	Interface - EBSCOhost Research Databases<br>Search Screen - Advanced Search<br>Database - APA PsycInfo	13,446

Scopus

((TITLE-ABS-KEY (construction AND (industr* OR sector* OR company* OR business* OR trade))) OR (TITLE-ABS-KEY (building AND (industr* OR sector* OR company* OR business* OR trade))) OR (TITLE-ABS-KEY ("construction worker" OR "civil engineer*" OR laborer* OR labourer* OR carpenter* OR electrician* OR builder* OR painter* OR plumber* OR "heavy equipment operator*" OR mason* OR pipefitter* OR "iron worker*"))) AND ((TITLE-ABS-KEY (work OR work AND place OR workplace OR organi?ation* OR worksite OR work AND site OR job AND site OR office)) AND (TITLE-ABS-KEY (intervention* OR program* OR prevention* OR training OR initiative* OR policy OR promotion OR campaign*))) AND ((TITLE-ABS-KEY (well-being OR wellbeing OR "mental wellbeing" OR "mental wellness" OR "psychological health" OR "psychological wellbeing" OR "psychological well-being" OR "psychological wellness" OR "mental health")) OR (TITLE-ABS-KEY (depress* OR "depress* symptoms" OR anxiety OR "anxiety symptoms" OR stress OR "stress symptoms" OR "job stress" OR "occupational stress" OR burnout OR burn-out))) AND (LIMIT-TO (PUBYEAR , 2020) OR LIMIT-TO (PUBYEAR , 2019) OR LIMIT-TO (PUBYEAR , 2018) OR LIMIT-TO (PUBYEAR , 2017) OR LIMIT-TO (PUBYEAR , 2016) OR LIMIT-TO (PUBYEAR , 2015) OR LIMIT-TO (PUBYEAR , 2014) OR LIMIT-TO (PUBYEAR , 2013) OR LIMIT-TO (PUBYEAR , 2012) OR LIMIT-TO (PUBYEAR , 2011) OR LIMIT-TO (PUBYEAR , 2010)) AND (LIMIT-TO (PUBSTAGE , "final")) AND (LIMIT-TO (DOCTYPE , "ar")) AND (LIMIT-TO (LANGUAGE , "English")) AND (LIMIT-TO (SRCTYPE , "j"))

Search History

Web of Science Core Collection

Set	Results	Save History / Create Alert / Open Saved History	Edit Sets
# 24	252	#19 AND #11 AND #10<br>**Refined by: PUBLICATION YEARS:** (2020 OR 2012 OR 2019 OR 2011 OR 2018 OR 2010 OR 2017 OR 2016 OR 2015 OR 2014 OR 2013) AND **LANGUAGES:** (ENGLISH) AND **DOCUMENT TYPES:** (ARTICLE) AND [excluding] **CONFERENCE TITLES:** (8TH INTERNATIONAL SEMINAR ON STRUCTURAL MASONRY FOR THE DEVELOPING COUNTRIES ISSM OR 9TH INTERNATIONAL CONFERENCE ON FIBRE REINFORCED POLYMER FRP COMPOSITES IN CIVIL ENGINEERING CICE OR CONFERENCE ON TUNNEL BORING MACHINES IN DIFFICULT GROUNDS TBM DIGS) AND [excluding] **Book Series Titles:** (ADVANCED STRUCTURED MATERIALS OR ROUTLEDGE SERIES ON COUNSELING WITH BOYS AND MEN OR SPON RESEARCH)<br>*Indexes=SCI-EXPANDED, SSCI, A&HCI, CPCI-S, CPCI-SSH, BKCI-S, BKCI-SSH, ESCI, CCR-EXPANDED, IC Timespan=All years*	
# 23	255	#19 AND #11 AND #10<br>**Refined by: PUBLICATION YEARS:** (2020 OR 2012 OR 2019 OR 2011 OR 2018 OR 2010 OR 2017 OR 2016 OR 2015 OR 2014 OR 2013) AND **LANGUAGES:** (ENGLISH) AND **DOCUMENT TYPES:** (ARTICLE) AND [excluding] **CONFERENCE TITLES:** (8TH INTERNATIONAL SEMINAR ON STRUCTURAL MASONRY FOR THE DEVELOPING COUNTRIES ISSM OR 9TH INTERNATIONAL CONFERENCE ON FIBRE REINFORCED POLYMER FRP COMPOSITES IN CIVIL ENGINEERING CICE OR CONFERENCE ON TUNNEL BORING MACHINES IN DIFFICULT GROUNDS TBM DIGS)<br>*Indexes=SCI-EXPANDED, SSCI, A&HCI, CPCI-S, CPCI-SSH, BKCI-S, BKCI-SSH, ESCI, CCR-EXPANDED, IC Timespan=All years*	
# 22	258	#19 AND #11 AND #10<br>**Refined by: PUBLICATION YEARS:** (2020 OR 2012 OR 2019 OR 2011 OR 2018 OR 2010 OR 2017 OR 2016 OR 2015 OR 2014 OR 2013) AND **LANGUAGES:** (ENGLISH) AND **DOCUMENT TYPES:** (ARTICLE)<br>*Indexes=SCI-EXPANDED, SSCI, A&HCI, CPCI-S, CPCI-SSH, BKCI-S, BKCI-SSH, ESCI, CCR-EXPANDED, IC Timespan=All years*	
# 21	329	#19 AND #11 AND #10<br>**Refined by: PUBLICATION YEARS:** (2020 OR 2012 OR 2019 OR 2011 OR 2018 OR 2010 OR 2017 OR 2016 OR 2015 OR 2014 OR 2013)<br>*Indexes=SCI-EXPANDED, SSCI, A&HCI, CPCI-S, CPCI-SSH, BKCI-S, BKCI-SSH, ESCI, CCR-EXPANDED, IC Timespan=All years*	
# 20	431	#19 AND #11 AND #10<br>*Indexes=SCI-EXPANDED, SSCI, A&HCI, CPCI-S, CPCI-SSH, BKCI-S, BKCI-SSH, ESCI, CCR-EXPANDED, IC Timespan=All years*	Edit
# 19	124,463	#18 OR #17 OR #6<br>*Indexes=SCI-EXPANDED, SSCI, A&HCI, CPCI-S, CPCI-SSH, BKCI-S, BKCI-SSH, ESCI, CCR-EXPANDED, IC Timespan=All years*	Edit
# 18	6,165	**TOPIC:** ("building industry" or "building sector*" OR "building company*" OR "building trade*")<br>*Indexes=SCI-EXPANDED, SSCI, A&HCI, CPCI-S, CPCI-SSH, BKCI-S, BKCI-SSH, ESCI, CCR-EXPANDED, IC Timespan=All years*	Edit
# 17	21,362	**TOPIC:** ("construction industry" or "construction sector*" OR "construction company*" OR "construction trade*")<br>*Indexes=SCI-EXPANDED, SSCI, A&HCI, CPCI-S, CPCI-SSH, BKCI-S, BKCI-SSH, ESCI, CCR-EXPANDED, IC Timespan=All years*	Edit
# 16	722	#11 AND #10 AND #7<br>**Refined by: PUBLICATION YEARS:** (2020 OR 2012 OR 2019 OR 2011 OR 2018 OR 2010 OR 2017 OR 2016 OR 2015 OR 2014 OR 2013) AND **DOCUMENT TYPES:** (ARTICLE) AND **LANGUAGES:** (ENGLISH) AND [excluding] **CONFERENCE TITLES:** (12TH INTERNATIONAL COASTAL SYMPOSIUM ICS OR 4TH INTERNATIONAL CONFERENCE ON UNDERSTANDING SMALL ENTERPRISES USE OR CONFERENCE ON TUNNEL BORING MACHINES IN DIFFICULT GROUNDS TBM DIGS OR 27TH EUROPEAN SYMPOSIUM ON COMPUTER AIDED PROCESS ENGINEERING ESCAPE OR 8TH INTERNATIONAL SEMINAR ON STRUCTURAL MASONRY FOR THE DEVELOPING COUNTRIES ISSM OR INTERNATIONAL CONFERENCE ON ACHEULEAN IN EUROPE ORIGINS EVOLUTION AND DISPERSAL OR 3RD EIASM WORKSHOP ON TALENT MANAGEMENT OR 9TH INTERNATIONAL CONFERENCE ON FIBRE REINFORCED POLYMER FRP COMPOSITES IN CIVIL ENGINEERING CICE)<br>*Indexes=SCI-EXPANDED, SSCI, A&HCI, CPCI-S, CPCI-SSH, BKCI-S, BKCI-SSH, ESCI, CCR-EXPANDED, IC Timespan=All years*	
# 15	730	#11 AND #10 AND #7<br>**Refined by: PUBLICATION YEARS:** (2020 OR 2012 OR 2019 OR 2011 OR 2018 OR 2010 OR 2017 OR 2016 OR 2015 OR 2014 OR 2013) AND **DOCUMENT TYPES:** (ARTICLE) AND **LANGUAGES:** (ENGLISH)<br>*Indexes=SCI-EXPANDED, SSCI, A&HCI, CPCI-S, CPCI-SSH, BKCI-S, BKCI-SSH, ESCI, CCR-EXPANDED, IC Timespan=All years*	
# 14	779	#11 AND #10 AND #7<br>**Refined by: PUBLICATION YEARS:** (2020 OR 2012 OR 2019 OR 2011 OR 2018 OR 2010 OR 2017 OR 2016 OR 2015 OR 2014 OR 2013) AND **DOCUMENT TYPES:** (ARTICLE)<br>*Indexes=SCI-EXPANDED, SSCI, A&HCI, CPCI-S, CPCI-SSH, BKCI-S, BKCI-SSH, ESCI, CCR-EXPANDED, IC Timespan=All years*	
# 13	1,016	#11 AND #10 AND #7<br>**Refined by: PUBLICATION YEARS:** (2020 OR 2012 OR 2019 OR 2011 OR 2018 OR 2010 OR 2017 OR 2016 OR 2015 OR 2014 OR 2013)<br>*Indexes=SCI-EXPANDED, SSCI, A&HCI, CPCI-S, CPCI-SSH, BKCI-S, BKCI-SSH, ESCI, CCR-EXPANDED, IC Timespan=All years*	
# 12	1,311	#11 AND #10 AND #7<br>*Indexes=SCI-EXPANDED, SSCI, A&HCI, CPCI-S, CPCI-SSH, BKCI-S, BKCI-SSH, ESCI, CCR-EXPANDED, IC Timespan=All years*	Edit
# 11	3,088,087	**TOPIC:**<br>(Well-being OR wellbeing OR “mental wellbeing” OR “mental wellness” OR “psychological health” OR “psychological wellbeing” OR “psychological well-being” OR “psychological wellness” OR “mental health” OR depress* OR “depress* symptoms” OR anxiety OR “anxiety symptoms” OR stress or “stress symptoms” OR “job stress” OR “occupational stress” OR burnout OR burn-out)	Edit

Indexes=SCI-EXPANDED, SSCI, A&HCI, CPCI-S, CPCI-SSH, BKCI-S, BKCI-SSH, ESCI, CCR-EXPANDED, IC Timespan=All years

# 10	802,438	#9 AND #8<br>*Indexes=SCI-EXPANDED, SSCI, A&HCI, CPCI-S, CPCI-SSH, BKCI-S, BKCI-SSH, ESCI, CCR-EXPANDED, IC Timespan=All years*	Edit
# 9	5,709,154	**TOPIC:** (intervention* OR program* OR prevention* OR training OR initiative* OR policy OR promotion OR campaign*)<br>*Indexes=SCI-EXPANDED, SSCI, A&HCI, CPCI-S, CPCI-SSH, BKCI-S, BKCI-SSH, ESCI, CCR-EXPANDED, IC Timespan=All years*	Edit
# 8	5,011,237	**TOPIC:** (work OR "work place" OR workplace OR organi?ation OR worksite OR "work site" OR "job site" OR office)<br>*Indexes=SCI-EXPANDED, SSCI, A&HCI, CPCI-S, CPCI-SSH, BKCI-S, BKCI-SSH, ESCI, CCR-EXPANDED, IC Timespan=All years*	Edit
# 7	316,653	#6 OR #5 OR #4<br>*Indexes=SCI-EXPANDED, SSCI, A&HCI, CPCI-S, CPCI-SSH, BKCI-S, BKCI-SSH, ESCI, CCR-EXPANDED, IC Timespan=All years*	Edit
# 6	99,527	**TOPIC:**<br>("construction worker" OR "civil engineer*" OR laborer* OR labourer* OR carpenter* OR electrician* OR builder* OR painter* OR plumber* OR "heavy equipment operator*" OR mason* OR pipefitter* OR "iron worker*")<br>*Indexes=SCI-EXPANDED, SSCI, A&HCI, CPCI-S, CPCI-SSH, BKCI-S, BKCI-SSH, ESCI, CCR-EXPANDED, IC Timespan=All years*	Edit
# 5	156,441	#3 AND #2<br>*Indexes=SCI-EXPANDED, SSCI, A&HCI, CPCI-S, CPCI-SSH, BKCI-S, BKCI-SSH, ESCI, CCR-EXPANDED, IC Timespan=All years*	Edit
# 4	89,100	#3 AND #1<br>*Indexes=SCI-EXPANDED, SSCI, A&HCI, CPCI-S, CPCI-SSH, BKCI-S, BKCI-SSH, ESCI, CCR-EXPANDED, IC Timespan=All years*	Edit
# 3	2,498,287	**TOPIC:** (industr* OR sector* OR company* OR business* OR trade)<br>*Indexes=SCI-EXPANDED, SSCI, A&HCI, CPCI-S, CPCI-SSH, BKCI-S, BKCI-SSH, ESCI, CCR-EXPANDED, IC Timespan=All years*	Edit
# 2	1,344,647	**TOPIC:** (building)<br>*Indexes=SCI-EXPANDED, SSCI, A&HCI, CPCI-S, CPCI-SSH, BKCI-S, BKCI-SSH, ESCI, CCR-EXPANDED, IC Timespan=All years*	Edit
# 1	776,788	**TOPIC:** (construction)<br>*Indexes=SCI-EXPANDED, SSCI, A&HCI, CPCI-S, CPCI-SSH, BKCI-S, BKCI-SSH, ESCI, CCR-EXPANDED, IC Timespan=All years*	Edit

Clarivate
Accelerating innovation

© 2021 Clarivate Copyright notice Terms of use Privacy statement Cookie policy

Sign up for the Web of Science newsletter Follow us
