# Supplementary material for: The effectiveness of organisational-level workplace mental health interventions on mental health and wellbeing in construction workers: A systematic review and recommended research agenda
Source: PLoS One. 2022 Nov 16;17(11):e0277114. doi: 10.1371/journal.pone.0277114 (PMC9668198; doi:10.1371/journal.pone.0277114)
Supplement: S2 File — (DOCX) [file pone.0277114.s003.docx]

**Outcomes of Full-Text Review**

| First Author | Year | Decision | Reason |
| --- | --- | --- | --- |
| Allahbaksh (1) | 2020 | Exclude | Wrong Study Design |
| Anger (2) | 2018 | Include | - |
| Ajayi (3) | 2019 | Exclude | Wrong Study Design |
| Campbell (4) | 2019 | Exclude | Wrong Outcome |
| Cedstrand (5) | 2020 | Exclude | Wrong Publication Type |
| Chen (6) | 2017a | Exclude | Wrong Study Design |
| Chen (7) | 2017b | Exclude | Wrong Outcome |
| Choi (8) | 2012 | Exclude | Wrong Outcome |
| De Silva (9) | 2017 | Exclude | Wrong Study Design |
| Dollard (10) | 2014 | Exclude | Wrong Population |
| Elo (11) | 2014 | Include | - |
| Guimaraes (12) | 2013 | Include | - |
| Gullestrup (13) | 2011 | Exclude | Wrong Outcome |
| Hensel (14) | 2010 | Exclude | Wrong Population |
| Jensen (15) | 2010 | Exclude | Wrong Outcome |
| Kent (16) | 2018 | Exclude | Wrong Study Design |
| King (17) | 2019 | Exclude | Wrong Outcome |
| Lange (18) | 2019 | Exclude | Wrong Population |
| Leung (19) | 2012 | Exclude | Wrong Outcome |
| Lingard (20) | 2015a | Exclude | Wrong Outcome |
| Lingard (21) | 2015b | Exclude | Wrong Study Design |
| Lingard (22) | 2017 | Exclude | Wrong Study Design |
| Lingard (23) | 2012 | Exclude | Wrong Outcome |
| Loudoun (24) | 2015 | Exclude | Wrong Outcome |
| Milner (25) | 2019 | Exclude | Wrong Publication Type |
| Nwaogu (26) | 2020 | Exclude | Wrong Study Design |
| Oude Hengel (27) | 2012 | Include | - |
| Oude Hengel (28) | 2013 | Include | - |
| Peters (29) | 2020 | Exclude | Wrong Outcome |
| Roach (30) | 2012 | Exclude | Wrong Population |
| Ross (31) | 2020 | Exclude | Wrong Outcome |
| Saeed (32) | 2017 | Exclude | Wrong Population |
| Sommovigo (33) | 2019 | Exclude | Wrong Study Design |
| Soni (34) | 2014 | Exclude | Wrong Outcome |
| Teimouri (35) | 2018 | Exclude | Wrong Study Design |
| Thomas (36) | 2020 | Exclude | Wrong Outcome |
| Vuori (37) | 2012 | Exclude | Wrong Population |

**References**

1. Allahbaksh S. Quality of work life of migrant workers in construction industry with focus on Chennai city. 2020;29(5).

2. Anger WK, Kyler-Yano J, Vaughn K, Wipfli B, Olson R, Blanco M. Total Worker Health Intervention for Construction Workers Alters Safety, Health, Well-Being Measures. J Occup Environ Med. 2018;60(8):700–9.

3. Ajayi SO, Jones W, Unuigbe M. Occupational stress management for UK construction professionals: Understanding the causes and strategies for improvement. J Eng Des Technol. 2019 Jan 1;17(4):819–32.

4. Campbell M.A. GJG. Strategies to improve mental health and well-being within the UK construction industry. 2020;173(2).

5. Cedstrand E, Nyberg A, Bodin T, Augustsson H, Johansson G. Study protocol of a co-created primary organizational-level intervention with the aim to improve organizational and social working conditions and decrease stress within the construction industry – a controlled trial. BMC Public Health. 2020 Dec;20(1):424.

6. Chen Y, McCabe B, Hyatt D. Impact of individual resilience and safety climate on safety performance and psychological stress of construction workers: A case study of the Ontario construction industry. J Safety Res. 2017 Jun;61:167–76.

7. Chen Y. MB Hyatt D. Relationship between Individual Resilience, Interpersonal Conflicts at Work, and Safety Outcomes of Construction Workers. 2017;143(8).

8. Choi SD. A study of trade-specific occupational ergonomics considerations in the U.S. construction industry. Work. 2012 Jun;42(2):215–22.

9. De Silva N, Samanmali R, De Silva H, De Silva N, Samanmali R, De Silva HL. Managing occupational stress of professionals in large construction projects. J Eng Des Technol. 2017;15(4):488–504.

10. Dollard MF, Gordon JA. Evaluation of a participatory risk management work stress intervention. Int J Stress Manag. 2014;21(1):27–42.

11. Elo A-L, Ervasti J, Kuosma E, Mattila-Holappa P. Effect of a leadership intervention on subordinate well-being. J Manag Dev. 2014 Jan 1;33(3):182–95.

12. Guimaraes L, Ribeiro J, Saurin T, de Bittencourt P, de Macedo Guimaraes LB, Duarte Ribeiro JL, et al. Circadian Rhythms as a Basis for Work Organization: A Study With Live Line Electricians. Hum FACTORS. 2013;55(1):204–17.

13. Gullestrup J, Lequertier B, Martin G, Gullestrup J, Lequertier B, Martin G. MATES in Construction: Impact of a Multimodal, Community-Based Program for Suicide Prevention in the Construction Industry. Int J Environ Res Public Health. 2011;8(11):4180–96.

14. Hensel J, Bender A, Bacchiochi J, Pelletier M, Dewa CS. A descriptive study of a specialized worker’s psychological trauma program. Occup Med. 2010 Dec 1;60(8):654–7.

15. Jensen L, Friche C, Jensen LK, Friche C. Implementation of New Working Methods in the Floor-Laying Trade: Long-Term Effects on Knee Load and Knee Complaints. Am J Ind Med. 2010;53(6):615–27.

16. Kent KB, Goetzel RZ, Roemer EC, McCleary K, Henke RM, Head MA, et al. Developing Two Culture of Health Measurement Tools: Examining Employers’ Efforts to Influence Population Health Inside and Outside Company Walls. J Occup Environ Med. 2018;60(12).

17. King T, Batterham P, Lingard H, Gullestrup J, Lockwood C, Harvey S, et al. Are Young Men Getting the Message? Age Differences in Suicide Prevention Literacy among Male Construction Workers. Int J Environ Res Public Health. 2019;16(3).

18. Lange S, Rowold J. Mindful leadership: Evaluation of a mindfulness-based leader intervention. Gr Interakt Organ Z Für Angew Organ GIO. 2019 Sep;50(3):319–35.

19. Leung M, Chan IYS, Yu J. Preventing construction worker injury incidents through the management of personal stress and organizational stressors. Accid Anal Prev. 2012 Sep;48:156–66.

20. Lingard H, Turner M, Charlesworth S, Lingard H, Turner M, Charlesworth S. Growing pains: work-life impacts in small-to-medium sized construction firms. Eng Constr Archit Manag. 2015;22(3):312–26.

21. Lingard, H. T M. Improving the health of male, blue collar construction workers: a social ecological perspective. 2015;33(1).

22. Lingard H. TM. Promoting construction workers’ health: a multi-level system perspective. 2017;35(5).

23. Lingard H, Francis V, Turner M. Work–life strategies in the Australian construction industry: Implementation issues in a dynamic project-based work environment. Int J Proj Manag. 2012 Apr;30(3):282–95.

24. Loudoun R. TK. Implementing health promotion programs in the Australian construction industry Levers and agents for change. 2017;24(2).

25. Milner A, King T, Scovelle A, Batterham P, Kelly B, LaMontagne A, et al. A blended face-to-face and smartphone intervention for suicide prevention in the construction industry: protocol for a randomized controlled trial with MATES in Construction. BMC PSYCHIATRY. 2019;19.

26. Nwaogu JM, Chan APC. Evaluation of multi-level intervention strategies for a psychologically healthy construction workplace in Nigeria. J Eng Des Technol [Internet]. 2020 Jan 1 [cited 2021 Mar 18];ahead-of-print(ahead-of-print). Available from: https://doi.org/10.1108/JEDT-05-2020-0159

27. Oude Hengel KM, Blatter BM, Joling CI, van der Beek AJ, Bongers PM. Effectiveness of an intervention at construction worksites on work engagement, social support, physical workload, and need for recovery: results from a cluster randomized controlled trial. BMC Public Health. 2012 Nov 21;12:1008.

28. Oude Hengel KM, Blatter BM, van der Molen HF, Bongers PM, van der Beek AJ. The effectiveness of a construction worksite prevention program on work ability, health, and sick leave: Results from a cluster randomized controlled trial. Scand J Work Environ Health. 2013;39(5):456–67.

29. Peters SE, Trieu HD, Manjourides J, Katz JN, Dennerlein JT. Designing a Participatory Total Worker Health® Organizational Intervention for Commercial Construction Subcontractors to Improve Worker Safety, Health, and Well-Being: The “ARM for Subs” Trial. Int J Environ Res Public Health. 2020 Jan;17(14):5093.

30. ROACH P, KEADY J, BEE P. “It’s easier just to separate them”: practice constructions in the mental health care and support of younger people with dementia and their families. J Psychiatr Ment Health Nurs John Wiley Sons Inc. 2012;19(6):555–62.

31. Ross V, Caton N, Gullestrup J, Kolves K, Ross V, Caton N, et al. A Longitudinal Assessment of Two Suicide Prevention Training Programs for the Construction Industry. Int J Environ Res Public Health. 2020;17(3).

32. Saeed S, Quock R, Lott J, Kashani N, Woodall W. Building Resilience for Wellness: A Faculty Development Resource. MedEdPORTAL. 2017;

33. Sommovigo V, Setti I, Maiolo M, Argentero P. Tunnel construction workers’ well-being: the role of job control and supervisor support. Int J Constr Manag. 2019;

34. Soni R. All in the mind? Saf Health Pract. 2014;32(2):40–1.

35. Teimouri H, Hosseini SH, Ardeshiri A. The role of ethical leadership in employee psychological well-being (Case study: Golsar Fars Company). J Hum Behav Soc Environ. 2018;28(3):355–69.

36. Thomas E, du Plessis M, Thomas K, Thomas EC, du Plessis M, Thomas KGF. An evaluation of job crafting as an intervention aimed at improving work engagement. SA J Ind Psychol. 2020;46.

37. Vuori J, Toppinen-Tanner S, Mutanen P. Effects of resource-building group intervention on career management and mental health in work organizations: Randomized controlled field trial. J Appl Psychol. 2012;97(2):273–86.
